# Machine learning modeling for identifying predictors of unmet need for family planning among married/in-union women in Ethiopia: Evidence from performance monitoring and accountability (PMA) survey 2019 dataset

**DOI:** 10.1371/journal.pdig.0000345

**Published:** 2023-10-17

**Authors:** Shimels Derso Kebede, Daniel Niguse Mamo, Jibril Bashir Adem, Birhan Ewunu Semagn, Agmasie Damtew Walle

**Affiliations:** 1 Department of Health Informatics, School of Public Health, College of Medicine and Health Science, Wollo University, Dessie, Ethiopia; 2 Department of Health Informatics, College of Medicine and health sciences, Arba Minch University, Arba Minch, Ethiopia; 3 Department of Public Health, College of Medicine and Health Science, Arsi University, Asella, Ethiopia; 4 Department of Public Health, School of Public Health, Asrat Woldeyes Health Science College, Debre Berhan University, Debre Berhan, Ethiopia; 5 Department of Health Informatics, Institute of Public Health, College of Medicine and Health Sciences, Mettu University, Mettu, Ethiopia; Tsinghua University, CHINA

## Abstract

Unmet need for contraceptives is a public health issue globally that affects maternal and child health. Reducing unmet need reduces the risk of abortion or childbearing by preventing unintended pregnancy. The unmet need for family planning is a frequently used indicator for monitoring family planning programs. This study aimed to identify predictors of unmet need for family planning using advanced machine learning modeling on recent PMA 2019 survey data. The study was conducted using secondary data from PMA Ethiopia 2019 cross-sectional household and female survey which was carried out from September 2019 to December 2019. Eight machine learning classifiers were employed on a total weighted sample of 5819 women and evaluated using performance metrics to predict and identify important predictors of unmet need of family planning with Python 3.10 version software. Data preparation techniques such as removing outliers, handling missing values, handling unbalanced categories, feature engineering, and data splitting were applied to smooth the data for further analysis. Finally, Shapley Additive exPlanations (SHAP) analysis was used to identify the top predictors of unmet need and explain the contribution of the predictors on the model’s output. Random Forest was the best predictive model with a performance of 85% accuracy and 0.93 area under the curve on balanced training data through tenfold cross-validation. The SHAP analysis based on random forest model revealed that husband/partner disapproval to use family planning, number of household members, women education being primary, being from Amhara region, and previously delivered in health facility were the top important predictors of unmet need for family planning in Ethiopia. Findings from this study suggest various sociocultural and economic factors might be considered while implementing health policies intended to decrease unmet needs for family planning in Ethiopia. In particular, the husband’s/partner’s involvement in family planning sessions should be emphasized as it has a significant impact on women’s demand for contraceptives.

## Author summary

This study aimed to identify key predictors contributing to women’s unmet need for family planning in Ethiopia. Eight machine learning algorithms were trained, and the random forest model was the most accurate with an area under the curve score of 0.93 and an accuracy of 85%. The top ten predictors were identified using the SHAP feature importance method, with husband/partner disapproval, primary education, being from the Amhara region, having previously delivered in health facilities, and middle wealth quantile being key factors. Women living in lower household members also increased the likelihood of unmet need for family planning. Unmet need for family planning can lead to unintended pregnancies, negative health outcomes for the mother and child, and economic and social consequences. It can also affect population growth rate, as women may have more children than they would otherwise choose to have, putting strain on resources and causing economic instability. To effectively address unmet needs, women’s education level, number of households, residence region, and place of delivery must be considered. Emphasizing husband/partner involvement in family planning sessions is crucial, as it significantly impacts women’s demand for contraceptives.

## Background

An unmet need for family planning occurs when a woman of reproductive age, whether married or in a union, and not using any form of contraception even though she wants to stop or delays childbearing [1]. Unmet need is a rights-based criterion that assesses how well a country’s health system and social conditions are responsive to women’s stated preference to delay or limit births [2]. It also indicates the success of reproductive health programs in meeting the demand of women for family planning services.

The unmet need for contraceptives is a public health issue globally with a substantial impact on maternal and child health. Women who carry unintended pregnancies to term may be less likely to seek antenatal care and delivery assistance, leading to an increase in maternal mortality [3]. Furthermore, unwanted pregnancy children are less likely to be breastfed and more likely to be stunted than wanted children [4] which increases their risk of child mortality. Hence reducing unmet need reduces the risk of abortion or childbearing by preventing unintended pregnancy.

In 2017, Africa had the highest proportion of married or in-union women of reproductive age with an unmet need for family planning, at 44%. In contrast, all other regions had a lower proportion of women, with only 25% having an unmet need for family planning [5]. Moreover, more than one out of every ten married or in-union women worldwide has an unmet need for family planning; and one out of every five women has an unmet need for family planning in Africa. In Sub-Saharan Africa (SSA), the overall prevalence of unmet need for family planning was 23.70%, with unmet need for spacing and limiting accounting for 15.81% and 7.9%, respectively [6]. Various study findings also reported a high unmet need of family planning in Ethiopia [7–9]. According to the Ethiopian Demographic and Health Survey (EDHS) report, total demand for family planning is increasing over time, about 58% of currently married women aged 15–49 have indicated a demand for family planning in 2016 [10]. Thirty-six percent of currently married women use a contraceptive method, either to spacing (22%) or to limit births (14%). However, unmet need remains high, at 22.3%, with Addis Ababa (11%), and Oromia region (29%).

The unmet need for family planning is one of several frequently used indicators for monitoring family planning programs, including the Millennium Development Goals (MDG) of improving maternal health [11]. It is also continues to be one of the major indicators in the *Sustainable Development Goals (SDG)* to ensure universal access to sexual and reproductive healthcare services by 2030. Despite anticipated reductions in some regions, the global unmet need for family planning is expected to remain above 10% in 2030. The greatest reduction is expected in Eastern Africa, where unmet need is targeted to reduce from 22% to 16% in 2030 [5]. The Ethiopian government has also set a target to double the number of women getting access to family planning methods by 2025 [12], with the aim of reducing the unmet need for family planning from 22% to 17% by 2030. Identifying predictors of unmet need for family planning using advanced machine learning analysis is critical to achieving these international and national goals.

Unmet need is sometimes interpreted as evidence of a lack of access to contraceptive supplies due to supply constraints or financial costs [11]. However, there are numerous sociodemographic, maternal-related, and service-related predictors of why women do not use contraception, such as place of residence, women’s age, women and husband’s education, wealth status, age at cohabitation, health care decision-making, visited health facility in the last 12 months, hearing about family planning methods by media, parity, number of under-five children, household size, and having knowledge on family planning methods [6,13,14]. Previous studies have addressed the unmet need for family planning, most of them using EDHS 2016 survey data and applied classical statistical models such as logistic regression. This study aimed to identify novel insights into the correlates of unmet contraception needs by (a) analyzing the more recent 2019 Performance Monitoring for Action (PMA) study; (b) applying more flexible, nonlinear, machine learning methods to model the data; and (c) applying SHAP values [15] to identify important predictors.

## Methods

### Study design

The study was conducted using secondary data from PMA Ethiopia 2019 Cross-sectional Household and Female survey. PMA-Ethiopia is a five-year(2019–2023) project implemented in collaboration with Addis Ababa University, Johns Hopkins University, and the Federal Ministry of Health which is made up of three distinct study activities such as annual cross-sectional surveys of women aged 15–49 years, longitudinal surveys of women who are currently pregnant or have recently given birth, and annual service delivery point surveys of health facilities [16]. This cross-sectional survey was carried out from September 2019 to December 2019.

### Source and study population

All 15–49 aged married women or women living with a partner in Ethiopia.

### Sample size

The sample size in this analysis was weighted to adjust for non-responses and variations in the probability of selection. Furthermore, the sample used is limited to responses from women who were married or living with a partner during the survey. Thus, the analysis was restricted to a weighted sample of 5819 reproductive-age women.

### Study variables

#### Dependent variable

Unmet need of family planning was the dependent variable which was dichotomized into two categories such as ‘unmet need’ and ‘no unmet need’.

#### Predictor variables

The predictor for unmet need includes socio-demographic and economic characteristics such as residence, women’s age, region, education level, wealth status, and media access. Reproductive health and family planning service characteristics such as ever been pregnant, age at first sex, ever used family planning (FP) methods, ever delivered in a health facility(HF), partner/husband feeling about FP, knowing any contraceptive method available, and partner told not to use FP were also incorporated as predictors of unmet need of family planning.

#### Data processing and analysis

The general framework used in an existing literature based on Yufeng Guo’s 7 Steps of Machine Learning was used in this study to predict unmet need for family planning. The framework describes the seven steps in supervised machine learning, which are as follows: data collection, data preparation, model selection, model training, model evaluation, parameter tuning, and prediction [17, 18]. Machine learning (ML) algorithms were implemented in Python 3.10.2 using Jupyter Notebook through scikit-learn, and XGBoost packages.

#### Data source/collection

The dataset for this study is available on the PMA Survey website and can be obtained upon a formal request. A weighted sample of 5819 reproductive-age women is included in the data.

#### Data preparation/pre-processing

Data cleaning, feature engineering, and data splitting were among the data preparation techniques used in this study.

Data cleaning consists of detecting and removing outliers, handling missing values, and handling unbalanced categories of the outcome variable from the data. Missing values of independent variables in the dataset were imputed through ‘CALIBERrfimpute’ R package. CALIBERrfimpute is an imputation mechanism constructed using Multivariate Imputation by Chained Equations (MICE) and random forest that allows for multiple imputations by sampling from conditional distributions [19]. To avoid ML models biased toward the majority class (no unmet need in this study), the training data was balanced using Synthetic Minority Oversampling Technique (SMOTE) [20], a random oversampling technique. SMOTE works by creating synthetic examples (new observations) that resemble the minority class by interpolating between minority class samples in the feature space, rather than creating exact copies of existing examples. However, there is still an unavoidable chance of overlapping or similarity between synthetic and original samples after oversampling. To compare the similarity or difference in the distribution of the synthetic data generated by SMOTE and existing data, we have applied Kolmogorov-Smirnov (KS) test (the kstest function from scipy.stats). Based on Kolmogorov-Smirnov test results (KS test statistic of 0.71125, p-value = <0.001), we have strong evidence against the null hypothesis, indicating that the distributions of the synthetic and existing observations significantly differ.

One-Hot-Encoding techniques were implemented using the Pandas get_dummies method to encode categorical to dummy variables with each category as a separate variable coded as 0 or 1 to indicate presence and absence respectively. SHAP feature importance was applied to assess the relationship between the predictors and the outcome variable and select independent variables that have the highest importance to predict unmet need of family planning. Finally, the whole data was split into training and testing by random assigning of 80% of the data for model training and 20% for tuning the trained model. But a tenfold cross-validation method on the training data was used for training the models. Ten-fold divides all observations into 10 equal-sized groups of samples called folds and 9 folds are used to train the model, and then 1 fold is used for testing 10 times repeatedly. Hence, the ten-fold cross-validation performance measure is the average of the values computed in this loop.

#### Model selection

The prediction task for this study was binary classification since unmet need for family planning was a categorical variable with two categories such as “unmet need” and “no unmet need”. Hence popular classification algorithms such as logistic regression (LR), Random forest (RF), K-nearest neighbor (KNN), artificial neural network, support vector machine (SVM), Naïve Bayes, eXtreme gradient boosting (XGBoost), and AdaBoost were used for this analysis. We have applied eight machine learning algorithms as they have different strengths and weaknesses and comparing multiple models allows us to explore various algorithms and techniques, helping us select the most appropriate model for our problem domain.

#### Model training

The selected classifiers were trained with both balanced and unbalanced data and their performance was compared through tenfold cross-validation. After comparison, the best predictive model was selected, tuned its hyperparameters, and trained with balanced training data for the final prediction on unseen test data.

#### Model evaluation

Model evaluation was performed after the model has been trained to determine how well the model performed on previously unseen test data based on its learning. The performance of the selected classifiers was compared through popular metrics such as classification accuracy and area under receiver operating characteristic curve (AUC) score. Furthermore, the receiver operating characteristic (ROC) curve was used for visualizing the performance of ML models.

#### Hyperparameter tuning

First, all of the models were trained with the default hyperparameters defined by the scikit-learn package. After model selection, the hyperparameters of the best model were tuned through the Optuna framework [21]. Optuna works by formulating the hyperparameter optimization as a process of minimizing or maximizing an objective function (such as accuracy) that takes a set of hyperparameters as an input and uses the Bayesian framework to understand better the probability of the optimal values and avoid unnecessary computation for the combination of non-performing parameters in the search. This framework is more efficient and flexible than traditional hyperparameter tuning techniques such as grid search and randomized search which both take explicitly user-defined hyperparameters and optimize the model only by those hyperparameters.

#### Prediction

Prediction is applying the final trained model put into production for its intended goal where the outcome variable will be estimated based on selected predictors. In this study, unmet need for family planning was determined based on top predictors identified during the analysis. Hence, whether a woman’s demand for family planning will be met or not will be determined through the best-performing classifier with a specified accuracy.

### Model interpretation/explanation using Shapley Additive exPlanations (SHAP)

Shapley Additive exPlanations (SHAP) analysis is based on a game theory to explain/interpret the prediction of any machine learning model, either globally or locally [15]. In machine learning research, it’s rare to see explanations and interpretations of high-performing models (usually tree-based models) due to their “black-box” nature. The fundamental concept behind the SHAP analysis is to compute the marginal contribution of each predictor towards the outcome variable prediction result. For this study we have applied SHAP analysis for two purposes:

1. For feature selection as SHAP global interpretability offers a unified measure of feature importance based on Shapley values, computed by quantifying each feature’s contribution to the prediction and aggregating it across the whole population. Various researchers applied SHAP as a feature selection mechanism and findings demonstrate that machine learning with SHAP value feature selection method has better classification performance with model explainability [22–25].
2. To interpret the effect of each predictor on unmet need prediction by plotting the aggregate Shapley value of that particular feature for every sample. Here we can explain whether a particular feature increases or decreases a woman’s likelihood for unmet need for family planning.

Furthermore, a waterfall plot was used to explain the contributions of each feature towards the prediction of a positive class (i.e. unmet need). In the waterfall plot, the x-axis represents the probability of classifying a sample as the "unmet need" class, while the y-axis displays the independent variables along with their corresponding feature values for that specific sample. Each feature’s contribution is represented by a horizontal bar in the waterfall plot. Positive contributions (red bars) indicate that the feature increases the likelihood of the sample belonging to the positive class. Negative contributions (blue bars) suggest a decrease in the likelihood of the sample belonging to the positive class. By analyzing the SHAP waterfall plot, we can gain insights into the relative importance and directionality of different features in determining the classification outcome for a specific sample.

**Fig 1:**
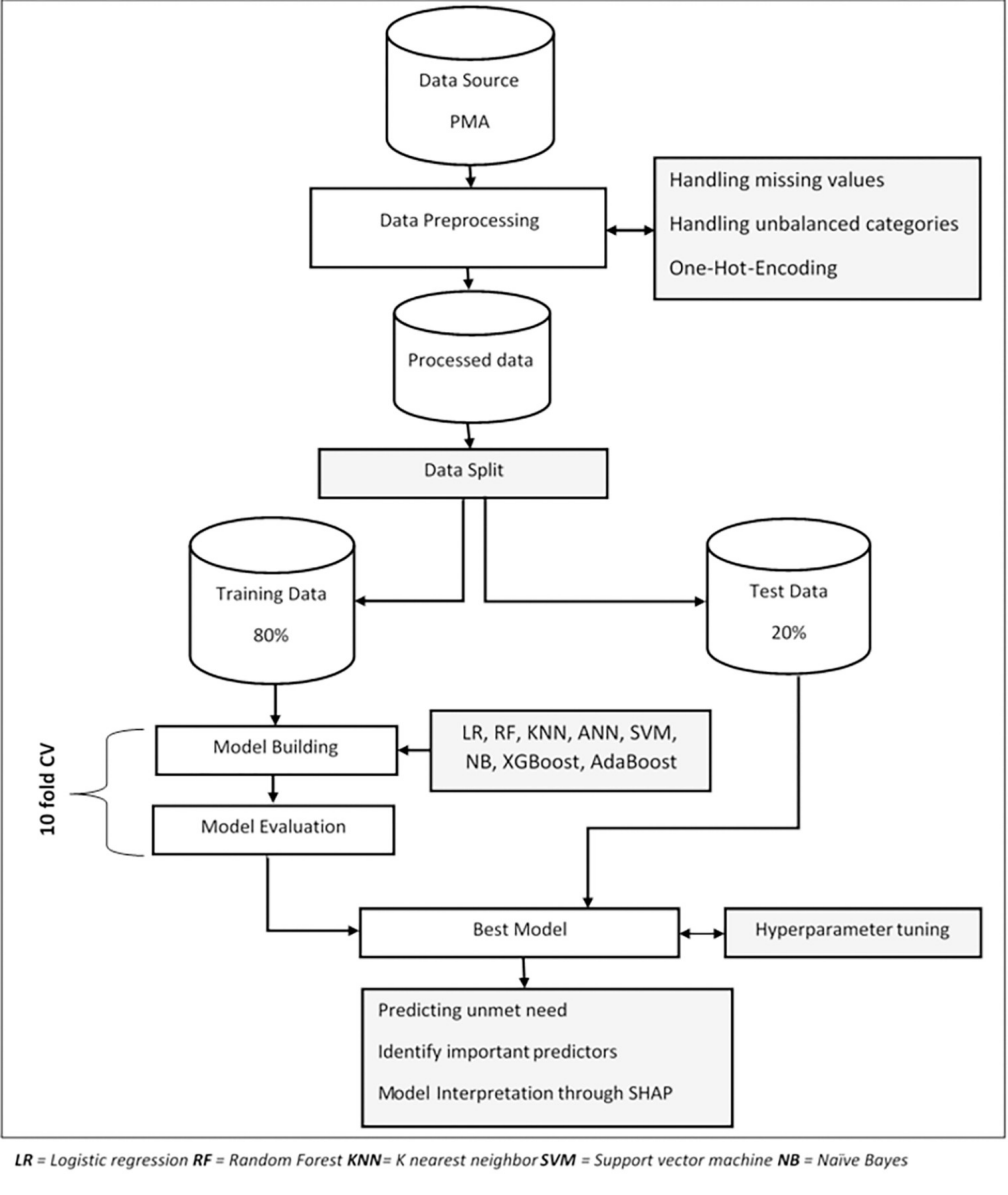
Overview flow chart of data preparation and analysis plan applied.

Finally, the overall data preparation and analysis process is presented in Fig 1.

### Ethics approval and consent to participate

Permission to use the data has been granted by the *PMA Ethiopia’s* survey project through legal registration. The data was used which is available on the public domain through the PMA website (https://www.pmadata.org/data/request-access-datasets) and can be accessed upon reasonable request after creating an account.

## Results

### Characteristics of participants

#### Socio-demographic and economic characteristics

Three-fourths (72.92%) of women were a rural residents and 2303 (39.57%) were in the age group between 26 and 35 years old. The majority of participants about 42.93% were poor, 48.12% were without education, and 62.94% had no media access. Regarding the regional distribution of respondents, the majority of the women were from Oromia (39.48%), followed by Amhara (23.08%), and about 19.83% from SNNPR. The remaining regions account for 17.61% of the total study population (Table 1).

**Table 1:** Socio-demographic and economic characteristics of respondents.

Variable	Categories	Weighted Freq.	Percent
Residence	Urban	1576	27.08
	Rural	4243	72.92
	15 to 25	1832	31.49
Woman’s age	26 to 35	2303	39.57
	36 to 49	1684	28.94
Region	Tigray	316	5.42
	Afar	75	1.29
	Amhara	1343	23.08
	Oromia	2297	39.48
	Somali	244	4.20
	Benishangul-Gumuz	65	1.11
	SNNPR	1154	19.83
	Gambela	22	0.37
	Harari	25	0.42
	Addis	252	4.33
	Dire Dawa	27	0.46
Education level	No education	2798	48.12
	Primary	2004	34.46
	secondary and above	1013	17.42
Wealth status	Poor	2498	42.93
	Middle	1144	19.66
	Rich	2177	37.41
Media access	No	3662	62.94
	Yes	2157	37.06

Number of household members

Mean = 5, Standard deviation = 2, Minimum: 1, Maximum = 16

#### Reproductive health and Family planning service characteristics

Among the total respondents, 5397 (92.75%) of them had a history of pregnancy, and about 4222(73.64%) women started sexual intercourse before the age of 18 years old. More than half of the respondents (54.40) had never used any family planning methods and about half of the women (49.13%) have never delivered in a health facility. Regarding their partner/husband feeling towards FP use, the majority of the women (62.60%) had the approval to use from their husband/partner (Table 2).

**Table 2:** Reproductive health and Family planning service characteristics of respondents.

Variable	Categories	Weighted Freq.	Percent
Ever been pregnant	No	422	7.25
	Yes	5397	92.75
Age at first sex	Before age 18	4222	73.64
	After age 18	1512	26.36
Ever used FP methods	No	1847	54.40
	Yes	1548	45.60
Ever delivered in HF	No	2572	49.13
	Yes	2663	50.87
Partner/husband’s feeling about FP	Disapproval	1420	25.55
	doesn’t care	659	11.85
	Approved	3480	62.60
know any contraceptive method available	No	73	1.25
	Yes	5746	98.75
Partner told not to use FP	No	4769	82.79
	Yes	992	17.21

### Machine learning analysis results

#### Balancing Data

The SMOTE oversampling technique generated 2782 additional synthetic observations from the minority category (i.e. unmet need) to balance the unbalanced distribution of the outcome variable. As a result, the overall distribution of unmet need status was changed from 873 unmet need, 3655 no unmet need; to give 3655 in each class to symmetric distribution for both categories for building reliable predictive models.

#### Model performance comparison

After model selection and training, the mean accuracy and mean area under the curve score of ML models in stratified 10-fold cross-validation were used to compare the performance of predictive models to predict unmet need for family planning. After comparing classifiers through stratified 10-fold cross-validation on the unbalanced training data, logistic regression was the best model with an accuracy of 80.7% and 0.63 area under the ROC curve. However, this result may not be reliable due to the unbalanced class nature of the outcome variable which may bias the model towards the majority class. To avoid this biased model building, ML model comparison was done after balancing the training data using SMOTE oversampling technique. Accordingly, Random Forest was the best predictive model with an accuracy of 84.8% and 0.92 area under the ROC curve (Table 3).

**Table 3:** Model comparison through tenfold cross-validation on training data.

ML Model	Performance	Unbalanced Data	Balanced data
Logistic Regression	Accuracy (%)	**80.7** ± **1.2**^**♦**^	72.4 ± 2.1
	AUC	**0.63 ± 0.05** ^**♦**^	0.79 ± 0.07
KNN	Accuracy (%)	78.3 ± 2.9	76.4 ± 3.2
	AUC	0.58 ± 0.08	0.86 ± 0.07
SVM	Accuracy (%)	80.6 ± 2.2	74.4 ± 3.1
	AUC	0.60 ± 0.04	0.79 ± 0.04
Random Forest	Accuracy (%)	79.6 ± 2.5	**84.8 ± 1.4** ^**♦**^
	AUC	0.62 ± 0.06	**0.92 ± 0.04** ^**♦**^
AdaBoost	Accuracy (%)	80.6 ± 3.4	71.9 ± 2.6
	AUC	0.62 ± 0.07	0.78 ± 0.05
XGBoost	Accuracy (%)	78.8 ± 3.5	81.5 ± 3.2
	AUC	0.62 ± 0.08	0.89 ± 0.06
Artificial Neural Net	Accuracy (%)	77.8 ± 4.1	78.5 ± 3.8
	AUC	0.62 ± 0.06	0.86 ± 0.04
Naïve Bayes	Accuracy (%)	71.3 ± 4.7	63.4 ± 5.2
	AUC	0.60 ± 0.09	0.71 ± 0.05

^**♦ =**^ Maximum performance

#### Hyperparameter tuning of Random Forest

Although scikit-learn provides a set of sensible default hyperparameters for all models including Random Forest, it is not guaranteed to be optimal for a problem. To maximize the performance of random forest, hyperparameters included the number of decision trees in the forest (n_estimators), the number of features considered by each tree when splitting a node(max_features), minimum number of samples required to split an internal node(min_samples_split), minimum number of samples required to be at a leaf node(min_samples_leaf), and number of samples to draw from independent variables to train each tree(max_samples) were optimized with one hundred trials on a given search space using stratified 10- fold cross-validation. The default hyperparameters set by scikit-learn and our optimized hyperparameters are shown in Table 4.

**Table 4:** Default and optimal tuned hyperparameters of Random Forest model.

Hyperparameter	Default	Optimal Value
**Number of trees**	100	125
**number of features considered for the best split**	Square root of the number of features	0.85
**minimum number of samples required to split an internal node**	2	4
**minimum number of samples required to be at a leaf node**	1	1
**number of samples to draw from X to train each base estimator**	None	1

Finally, random forest model was created with these tuned hyperparameters on balanced training data through 10-fold cross-validation and yielded 85% accuracy and 0.93 area under the curve.

#### Feature selection

**Fig 2:**
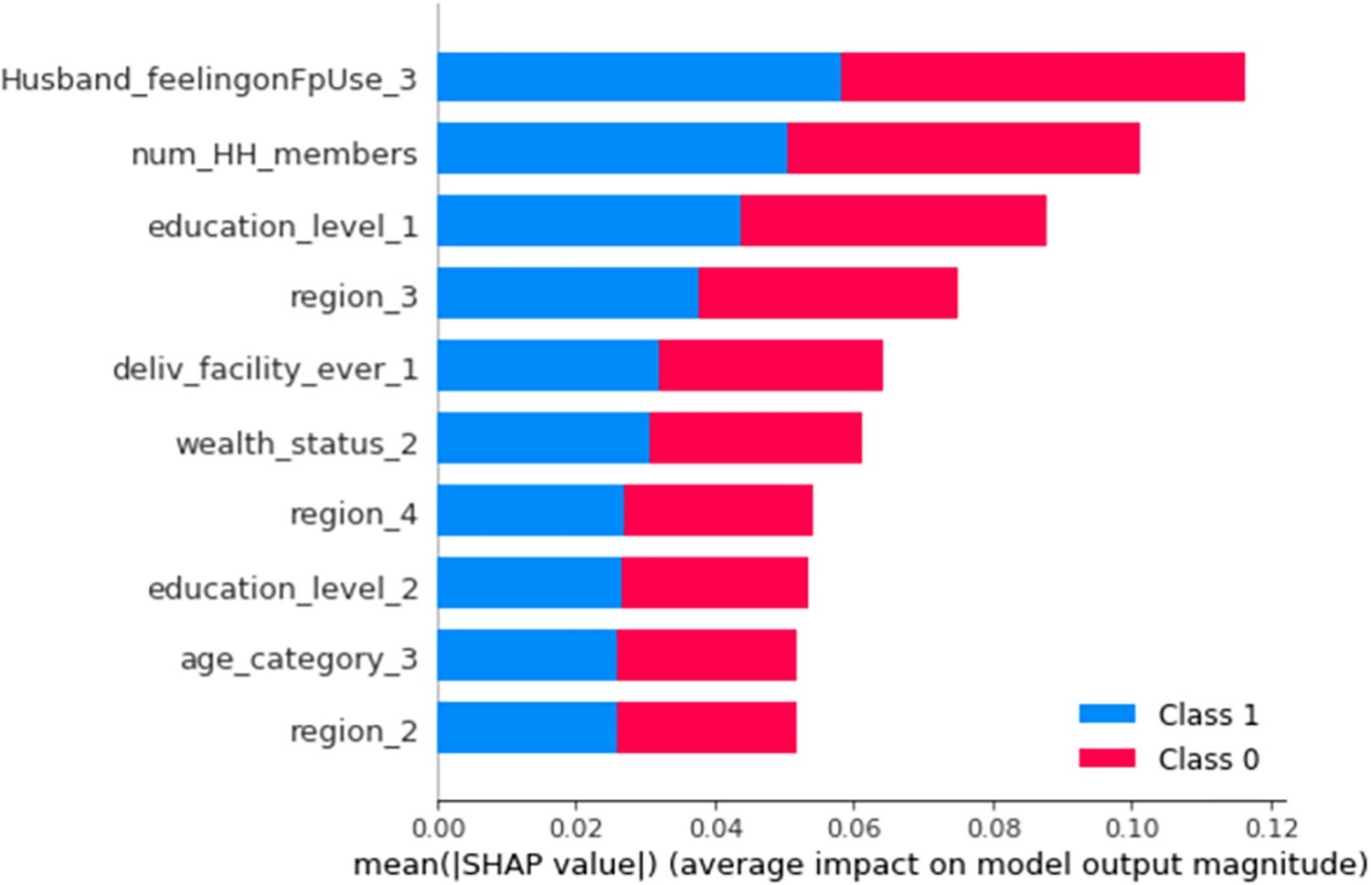
SHAP global importance plot of optimized Random Forest model. Husband_feelingonFpUse_3 = husband/partner disapproval to use family planning, num_HH_members = number of household members, education_level_1 = women education being primary, region_3 = Amhara region, deliv_facility_ever_1 = previously delivered in health facility, wealth_status_2 = middle wealth quantile, region_4 = Oromia region, education_level_2 = secondary and above women education, age_category_3 = 36 to 49 years old, region_2 = Afar region.

This study has used model agnostic SHAP global feature importance for selecting top predictors of unmet need for family planning. This technique examines the *mean absolute SHAP value* for each predictor across all of the data which quantifies the feature’s contribution towards the predicted unmet need status. Fig 2 illustrates the SHAP global importance scores for the top ten factors using the optimized random forest model. The predictors are sorted in descending order based on their impact on the outcome variable prediction and features with higher mean absolute SHAP values are more influential. The results revealed that the most important factors to predict unmet need for family planning are husband/partner disapproval to use family planning (Husband_feelingonFpUse_3), number of household members (num_HH_members), women education being primary (education_level_1), being from Amhara region (region_3), previously delivered in health facility (deliv_facility_ever_1) and middle wealth quantile (wealth_status_1) were the most important predictors of women’s unmet need for family planning. Furthermore, being from Afar (region_2) and Oromia (region_4), secondary and above women’s education (education_level _2), and age group 36 to 49 (age_category_3) were also important predictors of unmet need for family planning. As it is observable in the figure, the red and blue colors occupy half of the horizontal rectangles for each class. This means that each feature has an equal impact on the classification of both unmet need (label = 1) and no-unmet need (label = 0) cases (Fig 2).

#### Model interpretation/explanation

**Fig 3:**
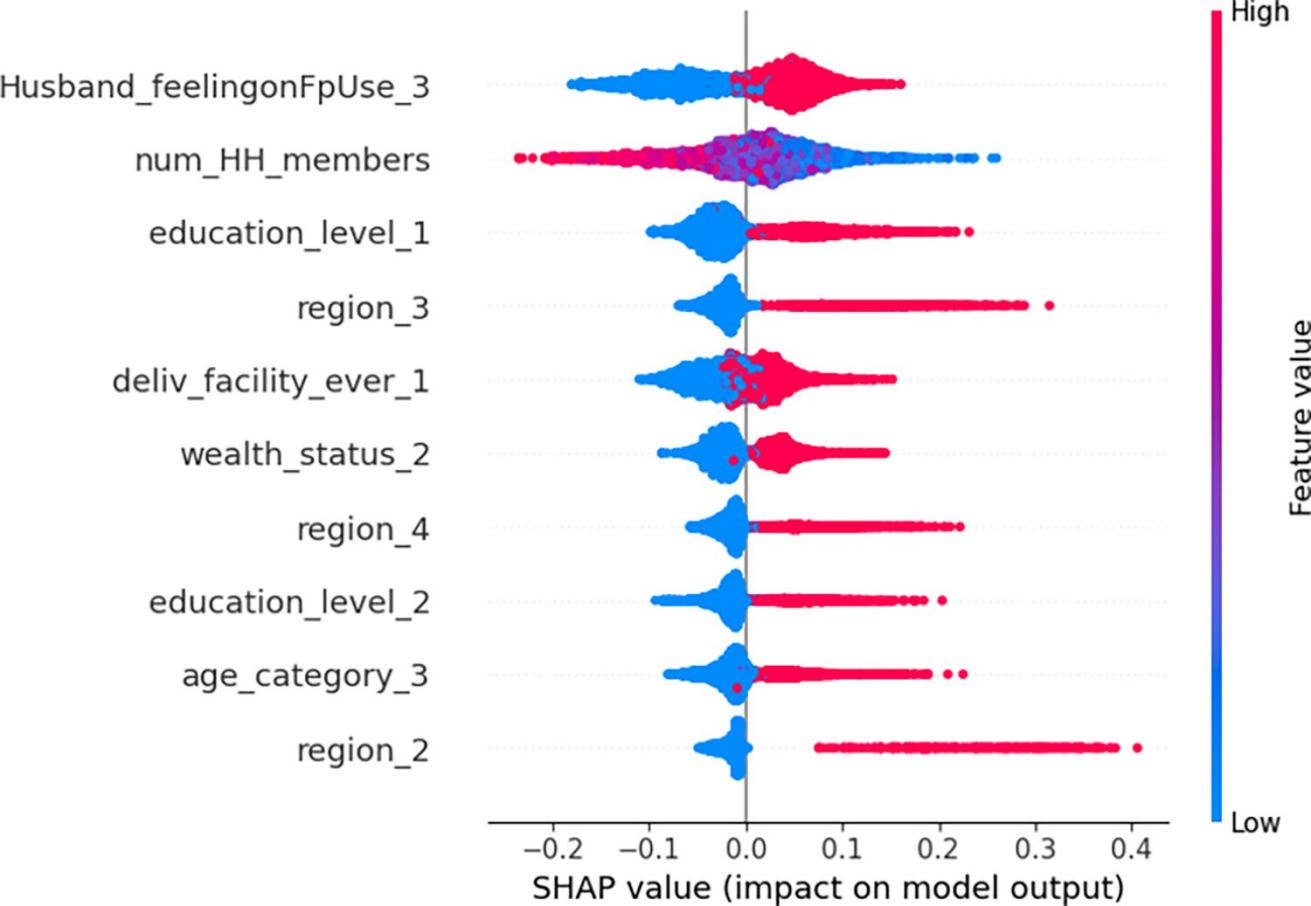
Beeswarm plot, ranked by mean absolute SHAP value generated by optimized Random Forest model. Husband_feelingonFpUse_3 = husband/partner disapproval to use family planning, num_HH_members = number of household members, education_level_1 = women education being primary, region_3 = Amhara region, deliv_facility_ever_1 = previously delivered in health facility, wealth_status_2 = middle wealth quantile, region_4 = Oromia region, education_level_2 = secondary and above women education, age_category_3 = 36 to 49 years old, region_2 = Afar region.

Beeswarms plots were used to provide a rich overview of how the variables impact the model’s predictions across all of the data. Fig 3 presents the distribution of the effects of each predictor on the model’s output (i.e. unmet need status prediction) by plotting the Shapley value of that particular predictor for every sample. The points on this beeswarm plot represent Shapley values of the features related to unmet need status, providing insight into the importance and association of each of the top ten features on the outcome variable. The red and blue hues in the figure represent the higher and lower values of each predictor’s variable. Points that are right to the vertical line (0 SHAP value) increase the likelihood of unmet need while the left side decreases unmet need likelihood (Fig 3). All variables except num_HH_members are categorical variables with two categories, so the red line represents category coded as 1 (high value) and the blue represents category coded 0 (low value). Hence husband/partner disapproval to use family planning (Husband_feelingonFpUse_3), women education being primary (education_level_1), being from Amhara region (region_3), previously delivered in a health facility (deliv_facility_ever_1) and middle wealth quantile (wealth_status_2), increases the likelihood of unmet need for family planning. Regarding the number of household members (num_HH_members), women living in a household with few members increases the likelihood of unmet need for family planning (Fig 3).

**Fig 4:**
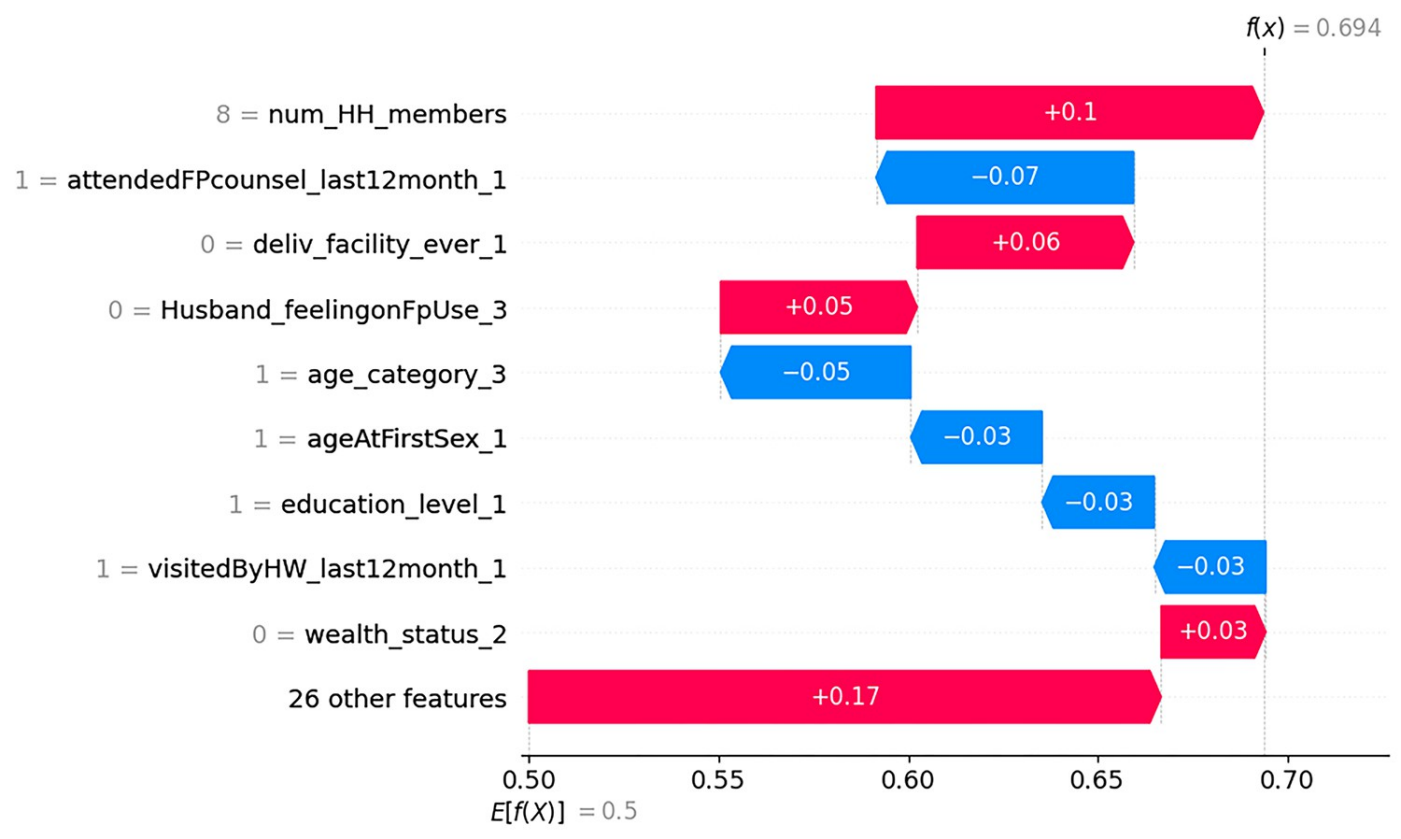
Waterfall plot displaying prediction of the first observation.

**Fig 5:**
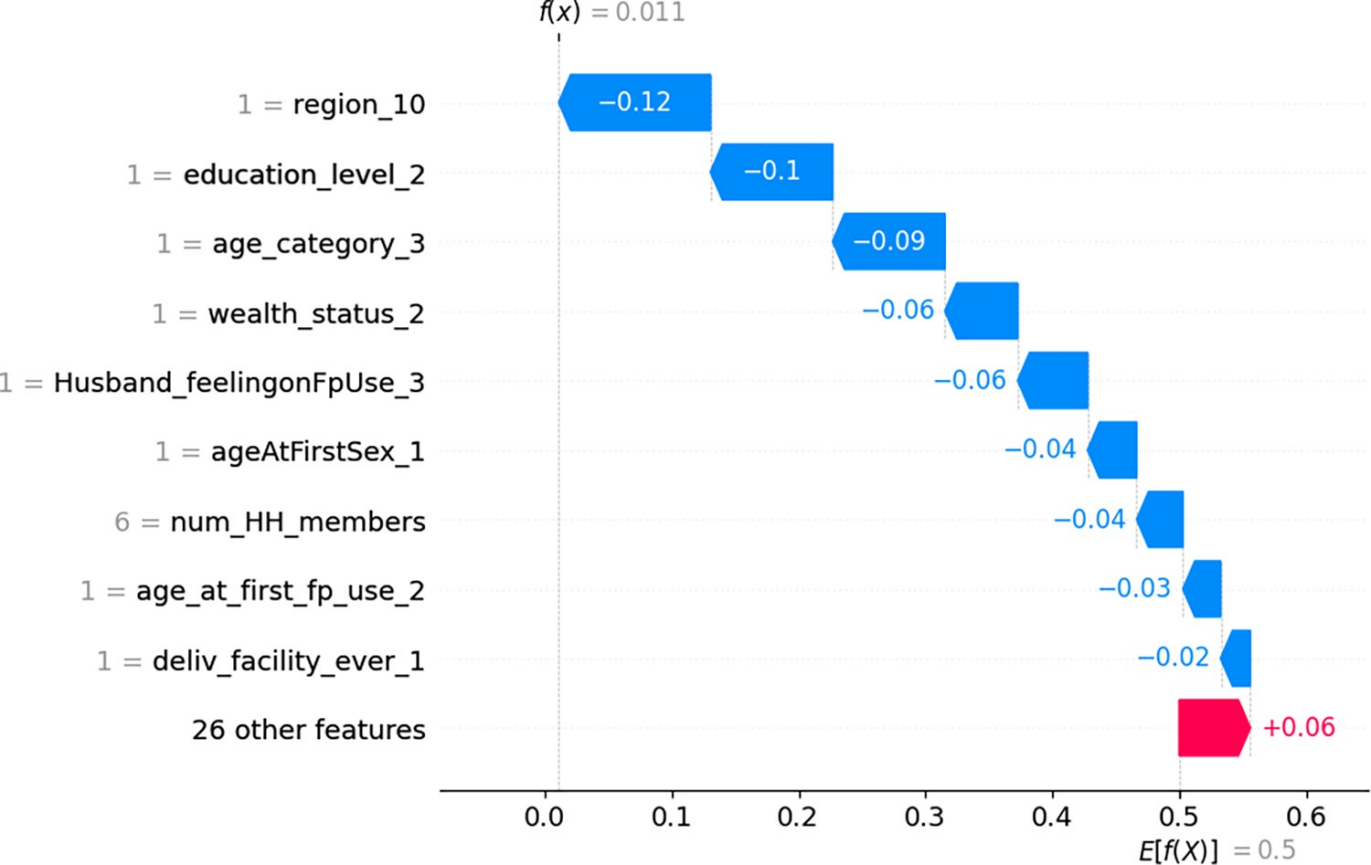
Waterfall plot displaying prediction of the second observation.

Finally, waterfall plots were used to explain the model prediction of the first and second observations in Fig 4 and Fig 5 respectively. In Fig 4, the waterfall plots begin with the expected value of the model output on the x-axis (*E*[*f*(*X*)] = 0.5), which represents the initial prediction for the given sample before considering any feature contributions. This baseline prediction is typically the average or most common prediction for the dataset.

For a given observation, if the model output above this value (*E*[*f*(*X*)]) corresponds to a positive class (i.e. unmet need) whereas scores below this value correspond to a negative class (“no unmet need”). Hence for the first observation, the combination of the positive contributions (in red) and the negative contributions (in blue) moves the expected value output to the final model output (*f*(*x*) = 0.69) classified as positive class (unmet need). Accordingly, having 8 members in the household (8 = num_HH_members), not delivering in a health facility (0 = deliv_facility ever_1), husband not approving family planning use (0 = husband_feelingonFpUse_3), and wealth status not being rich (0 = wealth_status_2) drives up the probability of having unmet need for family planning for this particular woman. Whereas attending family planning counseling in the last 12 months (1 = attendedFPcounsel_last12month_1), being aged 36 to 49 years old (1 = age_category_3), started sex after age of 18(1 = ageAtFirstSex_1), education level being primary (1 = education_level_1), and visited by health worker in the last 12 months (1 = visitedByHW_last12month_1) drives down the probability of this woman having unmet need for family planning (Fig 4).

Similarly, for the second observation, the combination of the positive contributions (in red) and the negative contributions (in blue) move the expected value output (E[f(x)] = 0.5) to the final model output (*f*(*x*) = 0.01) classified as positive class (unmet need). This means this observation has (1.0–0.001) 99% probability of not having an unmet need (negative class). For this particular woman, being from Addis Ababa (1 = region_10), secondary and above education level (1 = education_level_2), being aged 36 to 49 years old (1 = age_category_3), wealth status being rich (1 = wealth_status_2), husband approving family planning use (1 = husband_feelingonFpUse_3), started sex after age of 18 (1 = ageAtFirstSex_1), having 6 members in the household (6 = num_HH_members), starting use of family planning in the age of 21 to 30 years (1 = age_at_first_fp_use_2), delivering in health facility (1 = deliv_facility_ever_1) decreases the probability of having unmet need of family planning (Fig 5).

## Discussion

This study was conducted to identify key predictors of unmet need for family planning in Ethiopia. For this purpose, eight machine learning classifiers were trained on balanced and imbalanced training data through tenfold cross-validation. The performance of those eight classifier models was compared by their classification accuracy and AUC score. Consequently, models trained in data balanced through SMOTE resampling technique yielded higher accuracy and AUC score than models trained on imbalanced data. During the first phase of predictive modeling on unbalanced training data, logistic regression performed better than other classifiers with an accuracy of 80.7% and an AUC score of 0.63. However, Random Forest performed far better than logistic regression in the second phase of model building on balanced training data with an accuracy of 84.8% and 0.92 AUC score. Hence, Random Forest was the best predictive model and further analysis was performed after optimizing it for its optimal hyperparameters. The performance of hyperparameter-tuned Random Forest on balanced data was 85% accuracy and 0.93 area under the curve.

The SHAP analysis based on the RF model revealed that husband/partner disapproval to use family planning, number of household members, women education being primary, being from Amhara region, and previously delivered in a health facility were the top important predictors of unmet need for family planning in Ethiopia. Finally, SHAP model explanation was applied to interpret how each predictor affects the unmet need for family planning.

According to the SHAP model explanation husband/partner disapproval of women to use family planning increases the likelihood of unmet need for family planning. This finding was consistent with a study conducted in Toke Kutaye District, Oromia, Ethiopia[26] which reported that women whose husbands disapprove of the utilization of family planning were more likely to have an unmet need for family planning. Other studies in Ethiopia also reported that women whose partners had no supportive attitude toward family planning use were more likely to have an unmet need for family planning [27, 28]. This may be due to the husband’s/ partner’s lack of awareness, or myths and misinformation they hear about the side effects of family planning causing him to disapprove of his wife’s/partner’s use of family planning methods. Thus, strategies that involve male partners in contraceptive counseling sessions and engage men in reproductive health can improve contraceptive utilization while reducing unmet need by limiting husband’s disapproval of contraceptive use.

Living in households with fewer members increases the likelihood of women’s unmet need for family planning. This result was supported by findings from a study conducted in Pakistan that reported a higher likelihood of unmet need for family planning in households with a lower number of children [29]. It is also in line with other study’s findings in Pakistan[30] and Indonesia [31]. It indicates that with an increase in number of household members, women and other family members who can possibly be involved in fertility decisions may become more convinced regarding the usefulness of family planning methods.

This study also revealed that women’s education being primary increases the likelihood of unmet need for family planning. This result was in line with a study conducted in sub-Saharan Africa that reported a higher unmet need for both limiting and spacing among respondents who had completed primary education [6]. This could be due to women with only a primary education may be unaware of where contraceptive methods can be found and when to use them.

Another top predictor of unmet need for family planning was the region in which women living in. Those women who are from the Amhara region have a higher probability of unmet need for family planning. This finding was supported by various cross-sectional studies conducted in different parts of the Amhara region [27,32–34]. This is likely due to a combination of factors, including cultural norms, access to contraception, and the lack of education regarding family planning options since the majority of the respondents were rural residents.

This study also revealed that women who delivered in health facilities previously were more likely to have unmet need for family planning. The possible explanation for this could be women may not be given appropriate health information regarding family planning methods when seeking maternal services in health facilities. This also shows that facility delivery is not a guarantee that women’s demand for contraceptives will be met due to the multidimensional nature of the problem. Furthermore, evidence also showed substantial capacity gaps in the Ethiopian health system, challenging the provision of quality routine labor and delivery care services [35].

## Limitations and strengths of the study

The biggest challenge of using black-box machine learning models such as Random Forest is losing the ability to easily interpret the results and the factors driving the predicted outcomes. To mitigate the fact that machine learning results have interpretation limitations due to their black-box nature, the researchers used additional analyses to determine how predictors increased or decreased unmet contraceptive needs. The researchers used a combination of techniques such as SHAP to analyze the relative importance of each predictor and gain insight into how each factor contributed to the model’s predictions. This allowed them to better understand how the model’s predictions were affected by different factors.

While SHAP explanations offer valuable insights into individual predictions or cases, their ability to capture the overall behavior or patterns of a model is limited as the localized nature of SHAP explanations constrains their capacity to provide global understanding of models.

## Conclusions

The purpose of this study was to identify key predictors that contribute to women’s unmet need for family planning. For predicting unmet needs, eight machine learning algorithms were trained and their performance was evaluated according to their accuracy and area under the curve. Using tenfold cross-validation, the random forest model was the most accurate with an AUC of 0.93 and an accuracy of 85%. The top ten predictors of unmet need for family planning were identified through SHAP feature importance method, and their effects were explored. Finally, SHAP analysis identified husband/partner disapproval to use family planning, women education being primary, being from Amhara region, previously delivered in health facility, and middle wealth quantile key factors which increases the likelihood of unmet need for family planning. Furthermore, women living in lower number of household members increase the likelihood of unmet need for family planning. This shows that not only are certain characteristics of a woman (such as education, region, and wealth) associated with a higher likelihood of unmet need for family planning, but so are certain external factors, such as the disapproval of a husband or partner. This demonstrates the need for an approach that takes into account all these factors in order to effectively address unmet need for family planning. This is especially important because women who experience unmet need for family planning are more likely to experience unintended pregnancies, which can lead to negative health outcomes for both the mother and the child. Furthermore, unmet need for family planning can also have economic and social consequences, as it can lead to decreased economic opportunities for women and can exacerbate gender inequality. The unmet need for family planning can also have an effect on the population growth rate. When women are unable to access family planning services, they may have more children than they would otherwise choose to have. This can lead to rapid population growth, which can put a strain on resources and cause economic instability. Hence, women’s education level, the number of households, their residence region, and place of delivery must all be considered while implementing health policies intended to decrease unmet needs of family planning in Ethiopia. Moreover, the husband’s/partner’s involvement in family planning sessions should be emphasized as it has a significant impact on women’s demand for contraceptives.
